# Non-targeted metabolic profiling of BW312 *Hordeum vulgare* semi dwarf mutant using UHPLC coupled to QTOF high resolution mass spectrometry

**DOI:** 10.1038/s41598-018-31593-1

**Published:** 2018-09-04

**Authors:** Claire Villette, Julie Zumsteg, Hubert Schaller, Dimitri Heintz

**Affiliations:** 1 Plant Imaging and Mass Spectrometry, Institut de biologie moléculaire des plantes, CNRS, Université de Strasbourg, 12 rue du Général Zimmer, 67084 Strasbourg, France; 2 Plant Isoprenoid Biology, Institut de biologie moléculaire des plantes, CNRS, Université de Strasbourg, 12 rue du Général Zimmer, 67084 Strasbourg, France

## Abstract

Barley (*Hordeum vulgare*) is the fourth crop cultivated in the world for human consumption and animal feed, making it important to breed healthy and productive plants. Among the threats for barley are lodging, diseases, and pathogens. To avoid lodging, dwarf and semi-dwarf mutants have been selected through breeding processes. Most of these mutants are affected on hormonal biosynthesis or signalling. Here, we present the metabolic characterization of a brassinosteroid insensitive semi-dwarf mutant, BW312. The hormone profile was determined through a targeted metabolomics analysis by UHPLC-triple quadrupole-MS/MS, showing an induction of gibberellic acid and jasmonic acid in the semi-dwarf mutant. A non-targeted metabolomics analysis by UHPLC-QTOF-MS/MS revealed a differential metabolic profile, with 16 and 9 metabolites showing higher intensities in the mutant and wild-type plants respectively. Among these metabolites, azelaic acid was identified. Gibberellic acid, jasmonic acid, and azelaic acid are involved in pathogen resistance, showing that this semi-dwarf line has an enhanced basal pathogen resistance in absence of pathogens, and therefore is of interest in breeding programs to fight against lodging, but also probably to increase pathogen resistance.

## Introduction

Barley (*Hordeum vulgare*) is one of the major cereals cultivated in the world, along with maize, rice and wheat (www.fao.org/faostat). It has been domesticated several times in distinct regions spanning over the Fertile Crescent about 10,000 years ago^1,2^, which provided modern cultivars bearing exceptional agronomical traits regarding climate adaptation in different environments. Along with agriculture, barley evolved as a crop through natural selection, selective breeding, and the discovery of traits caused by spontaneous mutations, or by induction of random mutations by physical and chemical means to increase genetic variability. This process resulted in the selection of the phenotypes and the development of cultivars of interest^3^.

During the green revolution, dwarf and semi-dwarf lines have been selected because they are resistant to lodging, avoiding yield loss. Indeed, lodging happens during strong meteorological episodes, and leads to the fall of some plants, which are then difficult to harvest, and can pre-germinate or be contaminated by fungi^1,4^. At the genetic level, the green revolution found its genesis in the discovery of the *Rht* (*Reduced height*) gene in wheat^5^, isolated as loss of function allele encoding a negative regulator of gibberellin signalling^6^, and conferring a dwarf stature to the mutant plants. Following this discovery, other genes were investigated to induce a reduction in the plant size, resulting in a huge collection of gibberellin and brassinosteroid mutants, the most striking examples of plants displaying dwarf phenotypes^7,8^. Dwarf brassinosteroid mutants are also used in other crop species of agronomical importance as rice, pea and tomato^8^.

Part of the barley semi-dwarf mutants have been backcrossed to Bowman cultivar to facilitate their comparison and phenotyping, and form 57 independent near-isogenic lines referenced and available^9^. They are brassinosteroid mutants named after their phenotype, and ordered in groups: *brh* (*brachytic*), *ari* (*brevistarium*), *dsp* (*dense spike*), *ert* (*erectoides*), *uzu* (*semibrachytic*), *sdw* (*semidwarf*) and *sld* (*slender*). Two types of brassinosteroid mutants are described: biosynthesis mutants, which respond to exogenously applied brassinosteroids, and insensitive mutants, which do not. The analysis of the brassinosteroid content in these plants shows a deficiency in biosynthetic mutants, while insensitive mutants generally have an increased hormone content, associated with increased transcription level of brassinosteroid biosynthesis genes^8^. Brassinosteroid insensitive mutants of barley were first selected in Asia with the discovery of the *uzu* allele^10^, which was later introduced in cultivars of agricultural interest to confer a short and robust stature, rendering the plants resistant to lodging^1,11^. The insensitivity to exogenously applied brassinosteroid hormones is highlighted using the leaf unrolling assay^12^. First leaf segments of barley are incubated in distilled water in presence of brassinolide and kept in the dark for 72 h at 25 °C. Leaves from sensitive plants are unrolled in presence of the hormone, while leaves from brassinosteroid insensitive mutants stay rolled-up.

Among the barley brassinosteroid insensitive mutants is BW312, exhibiting a semi-dwarf phenotype due to a double substitution in the sequence coding for the brassinosteroid receptor BRI1 (brassinosteroid insensitive 1). This mutation is located on the kinase domain of the receptor which is essential for steroid binding, thus preventing the proper perception of the brassinosteroid signal in the plants. This was confirmed using the leaf unrolling assay, in which BW312 mutant showed a low sensitivity to exogenously applied 24-epi-brassinolide^11^. BW312 line was obtained by backcrossing the original *ert-ii.79* mutant (Bonus genetic background) to Bowman, and is characterized by a small stature, elongated basal internode, erect leaves and undulating leaf margins^13^. The castasterone level in BW312 mutant was shown to be higher than in its respective wild type Bowman^13,14^, which was also observed in brassinosteroid insensitive mutants of other species^15,16^. More recently, the original *uzu* barley line was also described as resistant to fungal and viral pathogens at a transcriptomic and biochemical level^17^. This enhanced pathogen resistance seems to be conserved in *bri1* mutants of *Brachypodium distachyon* inoculated with fungi^18^. However, to our knowledge, the metabolism at play in the establishment of a resistance phenotype has not been described so far.

Here, we go further in the characterization of the BW312 brassinosteroid insensitive mutant, using targeted and non-targeted approaches to describe the plantlets’ metabolome. Non-targeted metabolomics allows the differential comparison of biological material of interest. The samples were analysed by UHPLC-QTOF-MS/MS to identify differential metabolites distinguishing the brassinosteroid insensitive plantlets from the wild type ones. On the other hand, UHPLC-triple quadrupole-MS/MS was used to unveil the hormonal profile of two weeks old plantlets. This allowed us to describe a metabolic profile specific to the semi-dwarf plantlets that could explain the phenotype, maybe not only due to a hormonal disorder, but also to the reallocation of resources towards defence mechanisms.

## Results

**Figure 1:**
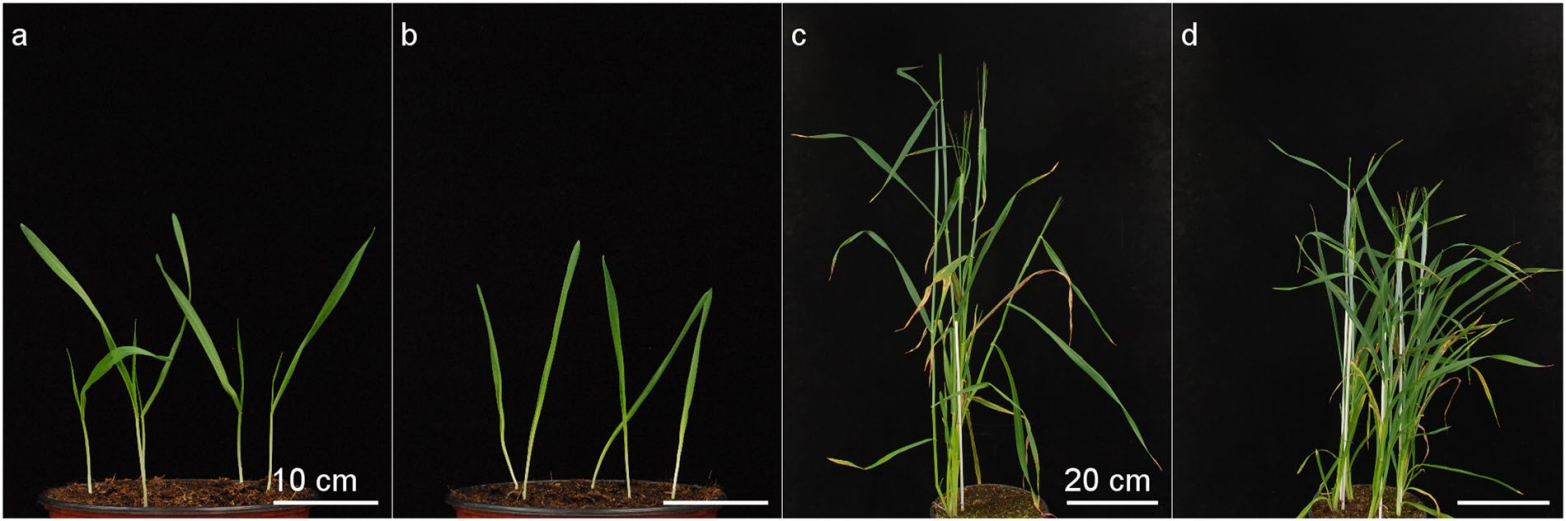
Phenotypes of the barley plants after 2 weeks of growth (**a**, Bowman; **b**, BW312) and three months of growth (**c**, Bowman; **d**, BW312). BW312 brassinosteroid insensitive mutant shows a late development after two weeks of growth and a reduced height at adult stage after three months of growth.

After two weeks of growth, a phenotypic difference could be observed between the wild-type line (Bowman) and the brassinosteroid insensitive mutant (BW312). The BW312 mutant plantlets showed one full leaf developed, while the wild-type plantlets had the second leaf emerging. Seven plantlets were sampled at this stage for extraction and metabolic analysis while others were left to grow to confirm phenotypes on older plants. At adult stage, the mutant displayed the expected semi-dwarf phenotype, as already described in the literature^13^ (Fig. 1).

### Non-targeted differential analysis

**Figure 2:**
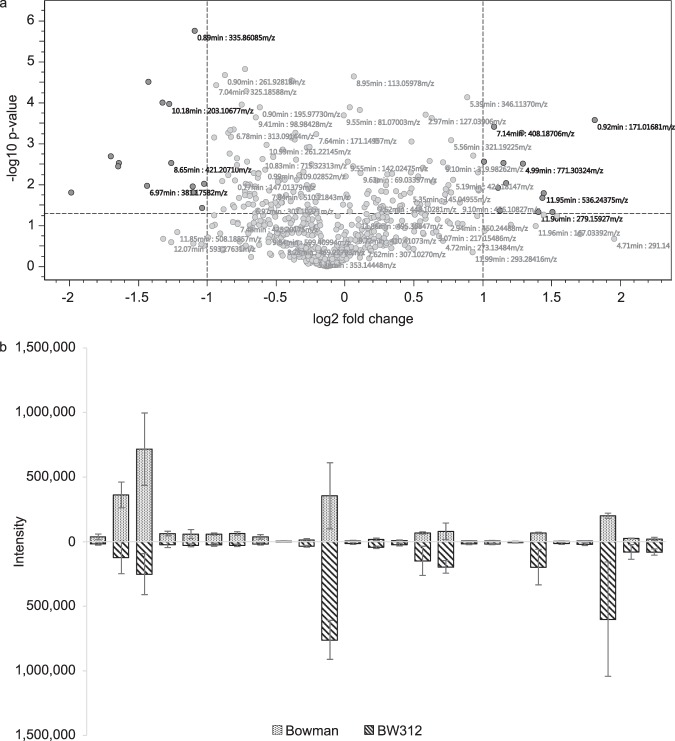
Comparative analysis of the buckets obtained from UHPLC-QTOF-MS in BW312 and Bowman plantlets extracts. Buckets are composed of a retention time and a m/z ratio describing one metabolite. Differential buckets were visualized with a volcano plot representation (**a**) before confirming the statistical difference using a Wilcoxon rank sum test. Differential buckets were present in both lines, in different intensities as represented in (**b**) upper part, intensities in Bowman samples; lower part, intensities in BW312 samples, expressed as mean values of 7 samples. Error bars represent standard deviation.

The metabolic profile of wild type and mutant plantlets was obtained using non-targeted analysis, which provided buckets composed of a measured m/z (mass/charge) ratio and a retention time, thus describing a molecule of interest. The buckets obtained in the two groups were compared to search for specific metabolites showing a higher intensity in one or the other group. This analysis showed 25 differential buckets using a Wilcoxon rank sum test (*p*-value < 0.05, fold change > 2): 9 were preferentially found in Bowman samples, and 16 in BW312 samples (Fig. 2a). These differential buckets were found in the two lines with different intensities (Fig. 2b). From the exact mass obtained in the buckets, a molecular formula was chosen using SmartFormula with a mSigma value lower than 20. The molecular formula was then used to interrogate Analyte DB, ChEBI, ChemSpider and PubChem databases through Compound Crawler tool available in Metaboscape 2.0 (Bruker Daltonics), leading to putative identifications. A molecular formula and a putative identification could be obtained for 6 buckets in Bowman and 6 buckets in BW312 (Table 1). The buckets for which no molecular formula and/or putative identification were found are given in Supplementary Table S1. In order to help further identification, a MS/MS analysis was performed on the mass of interest to obtain a mass spectrum, used in MetFrag to perform *in silico* fragmentation or to interrogate spectral libraries as described in the Methods section.

**Table 1:** Differential metabolites found in Bowman and BW312.

Retention time (min)	Measured m/z	mSigma	Molecular formula	Metfrag IntCov (%)	Name	Mode	Fold change BW312/Bowman	*p*-value
**Putatively identified**								
11.4	559.5199	2.64	C_36_H_66_N_2_O_2_	−	2,5-Dimethyl-3,6-bis(tetradecylamino)-1,4-benzoquinone	+	−3.620	0.004041
10.6	200.20149	6.96	C_12_H_25_NO	5.94	Dodecanamide	+	−2.856	0.006011
9.45	115.11185	2.71	C_7_H_14_O	25.053	2-Heptanone or 4-heptanone	+	−2.847	0.003034
10.17	221.11739	10.75	C_13_H_16_O_3_	55.226	(+)-(8R,8′R)-4-hydroxy-5-methoxy-1′,2′,3′,4′,5′,6′-hexanor-2,7′-cyclolignan-7′-one	+	−2.286	0.002676
10.18	203.10677	7.85	C_13_H_14_O_2_	68.564	Tremetone	+	−2.207	0.003948
8.65	421.2071	12.87	C_21_H_4_OS_4_	−	1-[(2-Methyl-2-propanyl)sulfanyl]-1-{tris[(2-methyl-2-propanyl)sulfanyl]vinyl}cyclopropane	+	−2.186	0.003948
6.12	102.09145	2.99	C_5_H_11_NO	−	Pentanamide	+	2.094	0.010128
1.59	97.02845	3.30	C_5_H_4_O_2_	−	Furfural	+	2.139	0.006011
9.13	244.19131	0.46	C_13_H_25_NO_3_	−	N-undecanoylglycine	+	2.391	0.006011
11.95	532.21237	12.59	C_32_H_29_N_5_OS	−	2-{4-[(4-Methyl-1-piperazinyl)methyl]phenyl}-7-[(4-phenoxyphenyl)amino]thieno[3,2-b]pyridine-6-carbonitrile	+	2.963	0.002700
0.92	171.01681	12.2	C_4_H_10_O_3_S_2_	−	7H-adenine;2-amino-3,7-dihydropurin-6-one	+	3.861	0.001745
**Identified**								
7.80	189.11259	7.80	C_9_H_16_O_4_	−	Azelaic acid	+	2.471	0.002676

Differential metabolites were determined using a Wilcoxon rank sum test with a *p*-value maximum set at 0.05. A molecular formula was generated for each metabolite of interest, with a maximum mSigma value of 20. When MS/MS was available, the putative identification was chosen using Metfrag *in silico* fragmentation. When MS/MS was not available, the putative identification was obtained by interrogation of libraries *via* Compound Crawler.

### Identification of differential metabolites

**Figure 3:**
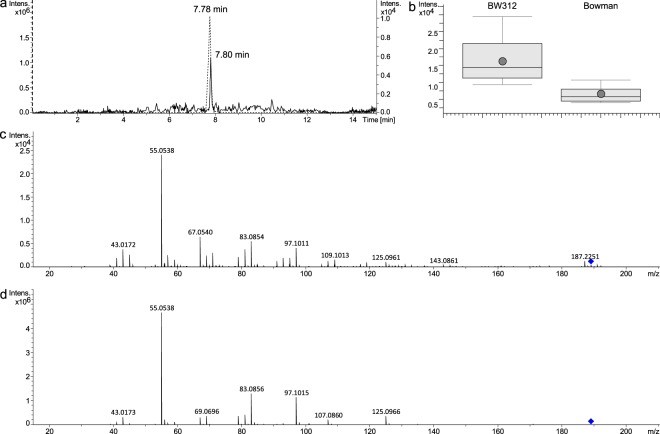
Identification of azelaic acid. The retention time and mass spectra of azelaic acid were obtained using an authentic standard. (**a**) EIC (extracted ion chromatogram) of azelaic acid is shown for a barley sample (straight line) and the standard (dashed line). (**b**) Higher intensities of azelaic acid were found in BW312 extracts than in Bowman (fold change 2.471). Mass spectra are given for a barley sample (**c**) and the standard (**d**).

When available, commercial standards of the putative metabolites were purchased and analysed with the same UHPLC-QTOF-MS/MS method to obtain their retention time and mass spectra. The standards were then implemented in a spectral library, and mass spectra of the buckets of interest could be compared to the spectra of the standards. Azelaic acid was confirmed after comparison of the mass spectra and retention time in the samples (7.80 min) and the standard (7.78 min) (Fig. 3a) and was present in higher amount in BW312 plantlets (Fig. 3b). The spectral library displayed a fit of 956.70 when comparing the spectra obtained from the MS/MS of precursor ion 189.11190 in samples and standard (Fig. 3c,d). Azelaic acid was identified in the samples with 11 fragments which could be assigned using *in silico* fragmentation provided by MetFrag tool (Supplementary Fig. S2). Standards of 2-heptanone, 4-heptanone, pentanamide and furfural were analysed but did not confirm the putative identifications.

### Targeted hormone analysis

To define the hormonal profile of the brassinosteroid insensitive plantlets, nineteen plant hormones were analysed in the methanolic extracts, belonging to different classes: brassinosteroids, auxins, cytokinins, gibberellins, strigolactones, jasmonates, salicylic acid, abscisic acid. Authentic standards were used to determine the retention time, collision energy and ionization mode to be used for the detection of the hormones of interest in the samples (Table 2).

**Table 2:** Parameters used for UHPLC-triple quadrupole-MS/MS analysis of plant hormones in MRM mode.

Hormone	Parent ion	Daughter ions	Retention time (min)	Collision energy (eV)	Mode	Hormone	Parent ion	Daughter ions	Retention time (min)	Collision energy (eV)	Mode
ABA	263	153.1	8.42	6	−	IBA	204	186.1	8.7	9	+
		219.1		9				130.1		25	
		204.1		18				144.1		23	
BA	123	87.1	7.8	7	+	JA	209	59.3	8.9	10	−
		105.1		3				41.4		30	
		69.1		13				109.1		16	
BR	481	238.7	10.45	8	+	JA ILE	322.2	130.2	9.72	18	−
		263		6				128.2		17	
		173.2		7				172.1		12	
cis-OPDA	293	275.1	10.6	7	+	KIN	216	41.3	6.2	8	+
		91.2		29				199.2		5	
		105.1		22				163.1		9	
CS	465	430	10.8	12	+	Oro	347	144.1	9.1	8	+
		448		5				204.1		6	
		385		16				161		22	
CT	433	397.3	11.5	8	+	SA	139	121	8	7	+
		415.4		5				69		13	
		379.2		14				45		12	
GA_1_	347	137.1	7.3	4	−	t-zea	220	136.1	5.1	14	+
		93.2		28				202		8	
								148		11	
GA_3_	345	143.1	7.2	23	−	2iP	204	109.1	7.2	14	+
		239.2		11				155		3	
		221.1		21				95.3		4	
GA_4_	331	225.2	9.6	36	−	^2^H_3_ CS	468	450.3	10.9	7	+
		269.1		36				432.2		13	
		243.1		36				458.8		6	
GA_7_	329	223.2	9.5	11	−	^2^H_6_ ABA	269	137.1	8.4	5	−
		283.3		5				93.2		30	
		45.4		75				121.1		6	
IAA	176	158.1	7.7	5	+						
		141.1		7							
		80.2		16							

The parameters used for hormone detection were defined using commercially available standards: parent and daughter ions, retention time, collision energy and ionization mode were adjusted for each hormone individually. ABA, abscisic acid; BA, benzoic acid; BR, brassinolide; cis-OPDA, cis-12-oxo-phytodienoic acid; CS, castasterone; CT, cathasterone; GA_1_, GA_4_, GA_7_, gibberellins A_1_, A_4_, A_7_; GA_3_, gibberellic acid; IAA, indole-3-acetic acid; IBA, indole-3-butyric acid; JA, jasmonic acid; JA ILE, jasmonoyl isoleucine; KIN, 6-furfurylaminopurine (kinetin); Oro, orobanchol; SA, salicylic acid; t-zea, trans zeatin; 2iP, 6-(γ,γ-dimethylallylamino)purine.

**Figure 4:**
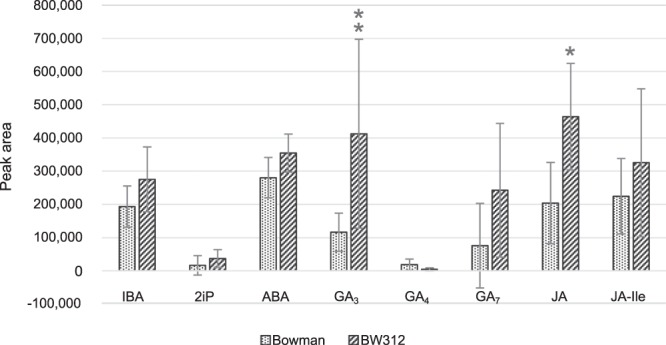
Hormones detected in BW312 and Bowman plantlets. The hormonal content of two weeks old plantlets was determined using UHPLC-MS/MS. Nineteen hormones of the different classes of plant hormones were searched for. Eight could be identified in the extracts, and the GA_3_ and JA contents were statistically different between the two barley lines. Hormonal content is expressed as area of the peaks obtained by UHPLC-MS/MS analysis. Statistical analysis (n = 7) was performed using a Wilcoxon rank sum test, *p*-value = 0.01107 (single asterisk) and 0.004079 (double asterisk). Error bars represent standard deviation. IBA, indole-3-butyric acid; 2iP, 6-(γ,γ-dimethylallylamino)purine; ABA, abscisic acid; GA_3_, gibberellin A_3_; GA_4_, gibberellin A_4_; GA_7_, gibberellin A_7_; JA, jasmonic acid; JA-Ile, jasmonoyl isoleucine.

Hormones were separated by liquid chromatography and identified according to their retention time, parent ion and daughter ions. Standards were added to a mix of the different samples to obtain the exact retention times in a matrix environment. CT, CS, BR, IAA, t-zea, SA, BA, Oro, GA_1_, cis-OPDA and KIN were not detected in the plantlets extracts but the standards were detected in the mix of standards and matrices used as a positive control, suggesting that these hormones were not present in the extracts or under the limit of detection. IBA, 2iP, ABA, GA_4_, GA_7_ and JA-ILE were detected in the samples, with no statistical difference between the peak areas when comparing BW312 samples to the Bowman ones. GA_3_ and JA were detected in the samples and presented a larger peak in BW312 extracts than in Bowman (Fig. 4), which was determined using a Wilcoxon rank sum test, with *p*-value = 0.004079 for GA_3_ and 0.01107 for JA. No difference was observed in the peak areas of the labelled CS and ABA, which shows that the extraction process had the same efficiency for the two lines.

After non-targeted analysis and hormonal profiling of the individual plantlets of both barley lines, the targeted analysis was repeated on concentrated samples, to get closer to the conditions used in published experiments (1 to 2 g fresh weight)^13,19^. This, to detect low abundant hormones such as brassinosteroids. In the concentrated extracts, we could detect castasterone, and confirm that its level was higher in the BW312 brassinosteroid insensitive mutant plantlets than in the wild type (Supplementary Fig. S3). Additionally, other hormones were detected such as cathasterone, indole butyric acid, salicylic acid, benzoic acid, and cis-OPDA (Supplementary Table S4). Azelaic acid was added to the list of targeted molecules to search for in the concentrated samples. The results also confirmed the accumulation of azelaic acid in the mutant plantlets using a targeted approach (Supplementary Fig. S3 and Table S4).

## Discussion

BW312 is a barley mutant which displays a dwarf phenotype due to brassinosteroid insensitivity. Indeed, this mutant carries a double substitution on the island domain of BRI1, preventing the binding of brassinosteroid hormones to the extracellular domain of the receptor. In brassinosteroid sensitive plants, brassinosteroid-associated kinase 1 (BAK1) acts as a co-receptor in the binding of brassinosteroids to BRI1, thus triggering the activation of BRI1 kinase domain, first step of a phosphorylation cascade leading to the expression of genes involved in cell elongation and plant growth (for review, see Belkhadir and Jaillais 2014)^20^. In fact, BAK1 can more generally act as a co-receptor of the family of leucine-rich repeat receptor like kinases (LRR-RLK). It is interesting to see that BAK1 interacts with flagellin sensitive 2 (FLS2) receptor, which is a sensor of bacterial flagellin peptide involved in the recognition of microbe-associated molecular patterns (MAMPs), and triggers plant defences *via* microbe-induced immunity (MTI). The recognition of MAMPs leads to the activation of FLS2 by association and transphosphorylation with BAK1, inducing the phosphorylation of the receptor-like cytoplasmic kinase BIK1 (botrytis induced kinase 1) and further transduction of the signal activating defence mechanisms and innate immunity^21^. In this study we show that brassinosteroid insensitive dwarf plantlets have increased levels of metabolites involved in plant stress responses (azelaic acid, GA_3_, JA), and seem to have increased basal defence mechanisms. These observations have already been made through pathogenicity tests in several species, proving that plant fitness depends on the equilibrium between growth and defence mechanisms. Indeed, *bri1* mutants of *Arabidopsis thaliana*, *Hordeum vulgare* and *Brachypodium distachyon* have been shown to be more resistant than their respective wild types in presence of viruses, pathogens or fungi^17,18,22,23^. However, the resistance was observed at a phenotypical and biochemical level but no clues were given concerning the metabolites involved in this enhanced resistance. One could expect that the blocking of brassinosteroid signalling leaves a larger pool of BAK1 available for FLS2 activation, but several studies proved it wrong, and highlighted a complex action of brassinosteroids on plant defence mechanisms. Indeed, Albrecht *et al*. 2012 showed that exogenous application of brassinosteroids on *Arabidopsis thaliana* leaves decreased MTI responses (oxidative burst and defence gene expression), but brassinosteroid signalling was not modified by induced MTI signalling. This happens without a decrease of FLS2-BAK1-BIK1 complex, suggesting that brassinosteroids can inhibit plant immunity downstream of the receptors^22^. This study also shows that BAK1 is not a rate-limiting factor for the two pathways. Belkhadir *et al*. 2012 underlines the importance of brassinosteroids homeostasis for MTI signalling, which is altered when the endogenous brassinosteroids level is increased or decreased^23^. This study shows that brassinosteroid modulation of MTI responses can occur through BAK1-dependent and independent mechanisms. In a more general point of view, brassinosteroids are well known to act on plant immunity, but the precise mechanisms involved are not yet deciphered. Opposite effects of brassinosteroids on defence responses were observed depending on the plant species, pathogen lifestyle and experimental conditions (whole plants, detached leaves, exogenous application or hormonal mutants…), which was well documented in a recent review^24^.

Azelaic acid (AzA) is involved in the priming of systemic acquired resistance (SAR) through the induction of salicylic acid (SA) production. SAR serves as a broad-spectrum protection for plants: mobile signals are produced at the site of primary infection after recognition of a pathogen, and translocated to distal tissues to allow rapid defence responses upon secondary infection^25^. AzA is one of these mobile signals, it accumulates in response to local wounding, and can be found systemically after infection including in healthy tissues. Jung *et al*. (2009) showed that AzA is found in petiole exudates, which application on *Arabidopsis thaliana* leaves could confer resistance to local and distal tissues^26^. Interestingly, JA was also found in petiole exudates and seems to be involved in long-distance information transmission to activate SAR responses^27^, before the accumulation of SA. This is consistent with the increased JA levels found in BW312 plantlets, along with increased levels of AzA. JA signalling would occur rapidly to set up SAR responses, while SA is accumulated later to establish and maintain systemic immunity. This hypothesis proposed by Truman *et al*. (2007) is compatible with the described fact that JA and SA responses are antagonistic. The presence of SA is necessary for SAR establishment but its accumulation is not required^26^, thus we are not surprised that SA could not be detected in the plantlets extracts, it may be under the limit of detection in extracts made from 15 mg dry weight. This is confirmed by the fact that SA was detected in concentrated samples, with a slight increase in BW312 extracts (Supplementary Table S4). Another metabolite which is known to act in priming plant immunity is pipecolic acid. Pipecolic acid was putatively identified in the buckets obtained from the non-targeted analysis, but no difference was found in the intensities between wild type and mutant plantlets, thus we did not try to confirm this putative identification.

Gibberellins (GAs) are plant hormones involved in myriad of physiological mechanisms leading to germination, growth, development, flowering and response to environmental conditions. GA signal induces the degradation of DELLA proteins that are nuclear growth-repressing proteins. After GA binding to GID1, the N-terminal domain of the receptor interacts with DELLA proteins to induce their degradation. GAs have been shown to act in plant immunity in a complex way as brassinosteroids do. In *Arabidopsis*, barley, wheat and rice, GAs could either induce or repress defence responses depending on the type of pathogen (biotrophs or necrotrophs)^24^. GAs and DELLAs are involved in the tuning of reactive oxygen species (ROS) production, acting on the induction of genes involved in ROS detoxification. The reduction of ROS levels decreases cell death, thus increasing diseases resistance^28^. Brassinosteroids have antagonistic effects on GA mediated immunity in rice by indirectly stabilizing DELLAs and the GA repressor slender rice 1 (SLR1). Exogenous treatment of rice plantlets with brassinolide decreased pathogen resistance^29^. The same observation was made using mutants: brassinosteroid-deficient mutants showed enhanced resistance compared to the wild types, and susceptibility was restored when the plantlets were treated with exogenous brassinolide. GA_3_ application on wild type plants enhanced pathogen resistance, while treatment with GA inhibitor promoted disease susceptibility. Interestingly, when treating the plants with both brassinolide and GA_3_, plants were susceptible again, while simultaneous treatment with brassinolide and GA inhibitor did not modify the plant susceptibility. Thus, De Vleesschauwer *et al*. (2012) proposed that GAs are essential for a full immune response to *P. graminicola* in rice^29^. The endogenous levels of the other gibberellins investigated in this study were not increased in comparison to the wild type.

In the frame of previously described enhanced pathogen resistance of *bri1* mutants in several model plants, here we investigate the metabolic signature of *bri1* barley plantlets. We show that in absence of brassinosteroid signalling, AzA, JA and GA_3_ levels are elevated in *bri1* barley plantlets, and correlate with a metabolic profile focused towards plant defence. In order to confirm these findings, we analysed the GA_3_, JA and AzA content of another *bri1* barley line that was already described as pathogen resistant^17^: *uzu1.a* in Bowman genetic background (BW885). In this study, BW885 was exposed to fungal and viral pathogens and exhibited a resistance phenotype. Grown in the same conditions and sampled at the same age (two weeks old) as BW312 plantlets, BW885 also showed higher levels of GA_3_ and JA and a slight increase in AzA content when compared to the control line (Supplementary Fig. S5). Therefore, BW312 plantlets show a similar metabolic profile to a pathogen resistant *bri1* barley line. It is to be noted that in our study, BW312 and BW885 plantlets were never subjected to pathogens. In this context, we propose that brassinosteroid insensitive plantlets of *Hordeum vulgare* BW312 mutant have an increased basal level of defence associated metabolites. The effective pathogen resistance of BW312 remains to be determined, but this barley line could be of interest for breeding purposes in order to bring semi-dwarfism and increased disease resistance, two features that are keys to higher yields.

## Methods

### Plant material

Seeds of barley lines Bowman, BW312 and BW885 were provided by the Carlsberg Research Laboratory (CRL, Copenhagen). The semi-dwarf mutant BW312 is a brassinosteroid signalling mutant. It exhibits a double substitution CC > AA at positions 1760 and 1761 of the gene coding for the brassinosteroid receptor HvBRI1^13,30^. These substitutions lead to the replacement of a threonine (T) by a lysine (L) at position 573 in the protein sequence. The mutation is situated on the island domain of the membrane-bound receptor that is necessary for brassinosteroid binding and further signal transduction. Therefore, BW312 mutant is insensitive to brassinosteroids and exhibits a semi-dwarf phenotype^13^. BW885 (uzu1.a) carries a single nucleotide substitution that leads to a His-857 to Arg-857 change in the kinase domain of BRI1^10^. Ten plants were cultivated in soil (Surfinia, Havita) for two weeks in cool conditions in a growth chamber under a 16 h light/8 h dark regime, at 18 °C during the light phase and 15 °C during the dark phase.

### Chemicals

Deionised water was filtered through Direct-Q UV (Millipore), methanol and isopropanol were purchased from Fisher Chemicals, NaOH from Agilent Technologies, acetic acid and formic acid from Sigma Aldrich. Standards were used to develop the UHPLC and MS/MS methods: abscisic acid (ABA, Sigma), benzoic acid (BA, Fluka), brassinolide (BR, OlChemIm), cis-12-oxo-phytodienoic acid (cis-OPDA, OlChemIm), castasterone (CS, OlChemIm), cathasterone (CT, OlChemIm), gibberellins A_1_, A_4_ and A_7_ (GA_1_, GA_4_, GA_7_ OlChemIm), gibberellic acid (GA_3_, Fluka), indole-3-acetic acid (IAA, Serva), indole-3-butyric acid potassium salt (IBA, Sigma), jasmonic acid (JA, OlChemIm), jasmonoyl isoleucine (JA ILE, OlchemIm), 6-furfurylaminopurine (kinetin, KIN, Duchefa), orobanchol (Oro, OlChemIm), salicylic acid (SA, Sigma), trans zeatin (t-zea, Sigma), 6-(γ,γ-dimethylallylamino)purine (2iP, Duchefa). ^2^H_3_ CS and ^2^H_6_ ABA were purchased from OlChemIm and used as internal standards spiked before extraction. Standards of 2-heptanone, 4-heptanone, pentanamide, furfural and azelaic acid were purchased from Sigma-Aldrich to confirm the identification of putative metabolites of interest obtained from the non-targeted analysis by UHPLC-QTOF-MS/MS.

### Sample preparation

After two weeks of growth, seven of the ten individuals were sampled, frozen in liquid nitrogen and immediately dried for 24 h. 15 mg of dry material were weighed in a 2 mL tube, and ground with metal beads for 4 minutes on a Tissue Lyser (Quiagen) at 30 Hz. The first extraction step was realized by pouring 1.5 mL of methanol complemented with ^2^H_3_ CS and ^2^H_6_ ABA on the dry plantlet powder. Samples were vortexed and shaken for 10 minutes at room temperature, and centrifuged for 5 minutes at 13,000 rpm, room temperature. The supernatant was transferred to a glass vial and evaporated with a SpeedVac concentrator (Savant SPD121P, Thermo Fisher) at room temperature. The pellets were extracted twice with 1.5 mL methanol, shaken, centrifuged and collected in the same glass vial to be evaporated. After 3 extractions, dry samples were solubilized in 100 µL of methanol. For non-targeted analysis, samples were diluted ten times in deionized water and placed in glass inserts for injection (Supplementary Fig. S6).

### Targeted hormone analysis

The hormone content of the plant extracts was analysed by ultra-performance liquid chromatography (UPLC) on Advance UHPLC (Bruker) coupled to EvoQ Elite (Bruker) mass spectrometer equipped with an electrospray ionization (ESI) source in MS/MS mode. The samples were kept at 4 °C before injection of 10 µL in full loop mode, and chromatographic separation on an Acquity UPLC ® HSS T3 C_18_ column (2.1 × 100 mm, 1.8 µm, Waters) coupled to an Acquity UPLC HSS T3 C_18_ pre-column (2.1 × 5 mm, 1.8 µm, Waters). Samples were carried through the column following a gradient of solvent A (H_2_O; 0.1% formic acid) and B (methanol; 0.1% formic acid). Chromatography was carried out at a flux of 0.300 mL.min^−1^, starting with 5% B for 2 minutes, reaching 100% B at 10 minutes, holding 100% B for 3 minutes and coming back to 5% B in 2 minutes, for a total run time of 15 minutes. The column was operated at 35 °C. Nitrogen was generated from pressurized air by Nitro 35 nitrogen generator (GeneGaz) and used as cone gas (30 L.h^−1^), heated probe gas (30 L.h^−1^) and nebulizing gas (35 L.h^−1^). The cone and heated probe temperatures were 350 °C and 300 °C respectively, and the capillary voltage was set at 3.5 kV. Hormones were analysed by multiple reaction monitoring (MRM), after determining the retention time and mode (positive or negative) by scan; and the cone voltage, daughter ion and collision energy using the MRM builder function on standards. Weak solvent (95% H_2_O, 5% MeOH) and strong solvent (95% methanol, 5% H_2_O) were used to wash the syringe. Results were processed using MS Data Review 8.2 (Bruker Daltonics) and checked manually. A mix of the different matrices spiked with the standards served as a positive control.

### Non-targeted metabolites analysis

Non-targeted metabolites analysis was performed on the UltiMate 3000 UHPLC system (Thermo) coupled to the ImpactII (Bruker) high resolution Quadrupole Time-of-Flight (QTOF). Chromatographic separation was achieved on an Acquity UPLC ® BEH C_18_ column (2.1 × 100 mm, 1.7 µm, Waters) coupled to an Acquity UPLC BEH C_18_ pre-column (2.1 × 5 mm, 1.7 µm, Waters), using the same chromatographic conditions as described for targeted hormone analysis. Injection was performed in a full loop mode with 20 µL of the samples. Calibration was set from 50 to 1000 Daltons (Da) using a fresh mix of 50 mL of isopropanol/water (50/50, v/v), 500 µL NaOH 1 M, 75 µL acetic acid and 25 µL formic acid. Samples were analysed in positive and negative MS modes at a spectra rate of 1 Hz, with capillary voltage set at 2.5 kV, nebulizer at 29 psi and dry gas at 8 L.min^−1^, with a dry temperature of 200 °C. Molecular features were determined using Data Analysis 4.4 (Bruker Daltonics), with a S/N ratio of 3, a correlation coefficient threshold set at 0.7 and a minimum compound length of 10 spectra. Bucket tables were generated from molecular features using Profile Analysis 2.3 (Bruker Daltonics), from m/z 50 to 1000 Da, using a value count of group attribute within bucket of 80%. The buckets of interest were identified and investigated in Metaboscape 2.0 (Bruker Daltonics) using a volcano plot representation, and then evaluating the statistical significance using a Wilcoxon rank sum test (*p*-value maximum set at 0.05). The molecular formula was generated using the Smart Formula tool available in Metaboscape 2.0, only formulae with a mSigma under 20 were chosen. Compound Crawler was interrogated from these formulae to generate putative identifications. The MS/MS spectra were obtained from a second injection of the samples after selection of the metabolites of interest (differential buckets). Mass spectra were then used in MetFrag to perform *in silico* fragmentation. Spectral libraries available in Metaboscape 2.0 and 3.0 were also interrogated: HMDB Metabolite Library (http://www.hmdb.ca/metabolites), Summer MetaboBASE Plant Library and Personal Library (https://www.bruker.com/products/mass-spectrometry-and-separations/ms-software/metabobase-personal-library/overview.html), MoNA library (http://mona.fiehnlab.ucdavis.edu/). Statistical analysis was performed in Metaboscape 3.0.

### Statistical analysis

For targeted hormone analysis, normality of the data was tested with Shapiro test on ANOVA residues, but regarding the small number of samples for each line (n = 7), a non-parametric approach was chosen for all statistics, even when normality was achieved. The Wilcoxon rank sum test was used in order to decide if the peak areas were statistically different between Bowman and BW312 extracts, with a maximum *p*-value set at 0.05.

For non-targeted analysis, considering the small number of individuals (n = 7), the Anderson-Darling test provided by Metaboscape 3.0 could not be used to determine the normality of the data. Therefore, the non-parametric Wilcoxon rank sum test was used to identify the buckets of interest regarding their intensity among the plant extracts. The *p*-value maximum was set at 0.05 and the fold change minimum set at 2 to determine which buckets were selected. Statistical analysis was made separately on the data acquired on positive and negative MS modes.

## Electronic supplementary material

Supplementary information

## Data Availability

The datasets generated and analysed during the current study are available from the corresponding author on reasonable request. Biological material is available in public seed banks as the International Database for Barley Genes and Barley Genetic Stocks (www.nordgen.org).
