# Supplementary material for: Non-targeted metabolic profiling of BW312 Hordeum vulgare semi dwarf mutant using UHPLC coupled to QTOF high resolution mass spectrometry
Source: Sci Rep. 2018 Sep 4;8:13178. doi: 10.1038/s41598-018-31593-1 (PMC6123459; doi:10.1038/s41598-018-31593-1)
Supplement: Supplementary file 1 — Supplementary information [file 41598_2018_31593_MOESM1_ESM.pdf]

Supplementary information

Non-targeted metabolic profiling of BW312 *Hordeum vulgare* semi dwarf mutant using UHPLC coupled to QTOF high resolution mass spectrometry.

Claire Villette[1]*, Julie Zumsteg[1], Hubert Schaller[2], Dimitri Heintz[1]

claire.villette@ibmp-cnrs.unistra.fr

julie.zumsteg@ibmp-cnrs.unistra.fr

hubert.schaller@ibmp-cnrs.unistra.fr

dimitri.heintz@ibmp-cnrs.unistra.fr

[1]Plant Imaging and Mass Spectrometry, Institut de biologie moléculaire des plantes, CNRS, Université de Strasbourg, 12 rue du Général Zimmer, 67084 Strasbourg, France.

[2]Plant Isoprenoid Biology, Institut de biologie moléculaire des plantes, CNRS, Université de Strasbourg, 12 rue du Général Zimmer, 67084 Strasbourg, France.

Supplementary Table S1. Differential buckets which could not be identified (no mass formula or putative identification).

RT (min)	Measured m/z	mSigma	Putative molecular formula	Fold change BW312/Bowman	*p*-value
**9.45**	249.20658	0.88	$C_{13}H_{28}O_4$	-2,968	0.008809
**6.97**	381.17582	2.07	$C_{16}H_{28}O_{10}$	-2,471	0.006642
**10.37**	98.98428	-	-	-2,456	0.001745
**1.54**	520.17739	4.37	$C_{20}H_{29}N_3O_{13}$	2,208	0.006485
**7.14**	408.18706	-	-	2,326	0.004041
**7.80**	171.10157	7.56	$C_9H_{14}O_3$	2,367	0.006642
**9.10**	403.19638	1.84	$C_{19}H_{30}O_9$	2,436	0.012716
**3.86**	422.16587	16.51	$C_{14}H_{19}N_{11}O_5$	2,669	0.002700
**4.99**	771.30324	7.97	$C_{31}H_{50}N_2O_{20}$	2,685	0.004275
**11.96**	279.15927	1.13	$C_{16}H_{22}O_4$	2,905	0.035006
**11.95**	536.24375	-	-	2,981	0.001745
**11.96**	391.28491	8.57	$C_{24}H_{38}O_4$	2,981	0.025347
**11.96**	261.14866	1.06	$C_{16}H_{20}O_3$	3,115	0.012716

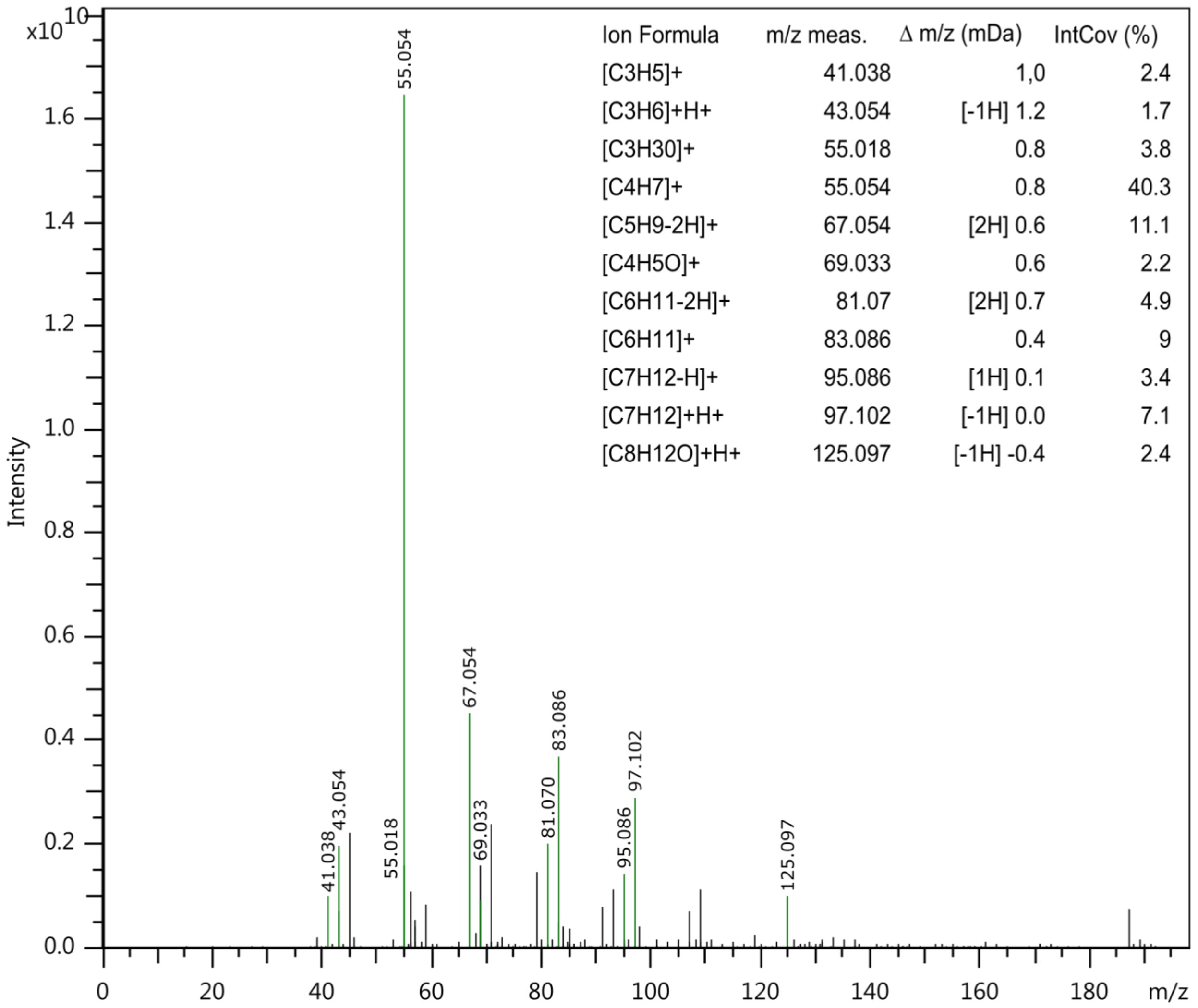

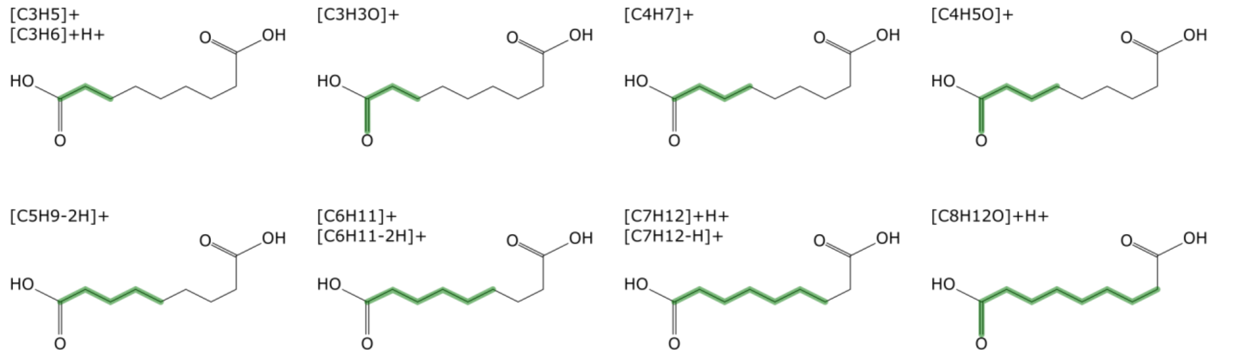

Supplementary Figure S2. *In silico* fragmentation of azelaic acid identified in barley samples. *In silico* fragmentation was perfomed using MetFrag, which returned 11 fragments that could be assigned to the azelaic acid structure (highlighted in green).

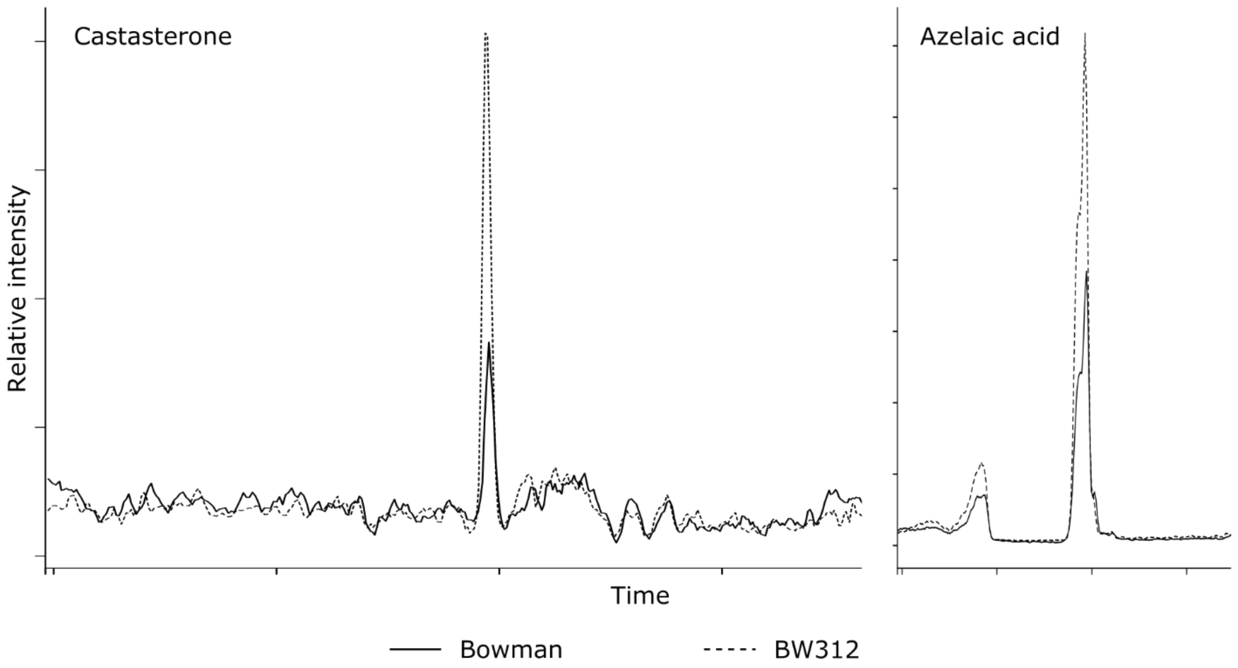

Supplementary Figure S3. Castasterone and azelaic acid detection in Bowman and BW312 concentrated samples. Targeted analysis was performed on the pooled concentrated samples (equivalent 2g fresh weight) in order to search for low abundant hormones. Castasterone was detected, and the intensity and peak area were higher in BW312 samples, as previously shown in the literature. We confirmed through a targeted approach the higher intensity of azelaic acid peak in the mutant plantlets.

Supplementary Table S4. Targeted analysis in concentrated samples (equivalent 2g fresh weight). The hormonal profile of concentrated samples was analysed in MRM mode using the same parameters as described in **Table 2**. Benzoic acid (BA), cis-12-oxo-phytodienoic acid (cis-OPDA), castasterone (CS), cathasterone (CT) and salicylic acid (SA) were identified in the concentrated extracts in addition to the other hormones identified in the non-concentrated samples (ABA, abscisic acid; GA_4, GA_7, gibberellins A_4 and A_7; IBA, indole-3-butyric acid; JA, jasmonic acid). We confirmed using a targeted analysis that the peak area for azelaic acid (AzA) in BW312 extract is higher than in Bowman.

Name	Area in Bowman	Area in BW312	Mode	Name	Area in Bowman	Area in BW312	Mode
**ABA**	475,084	884,638	-	**GA_4**	3,157	7,772	-
**AzA**	3,824,000	4,813,000	-	**GA_7**	2,238	3,072	-
**BA**	36,012	56,384	+	**IBA**	135,557	252,739	+
**cis-OPDA**	82,309	967,615	+	**JA**	199,529	423,215	-
**CS**	15,886	27,282	+	**SA**	577,785	817,310	+
**CT**	8,573	2,715	+				

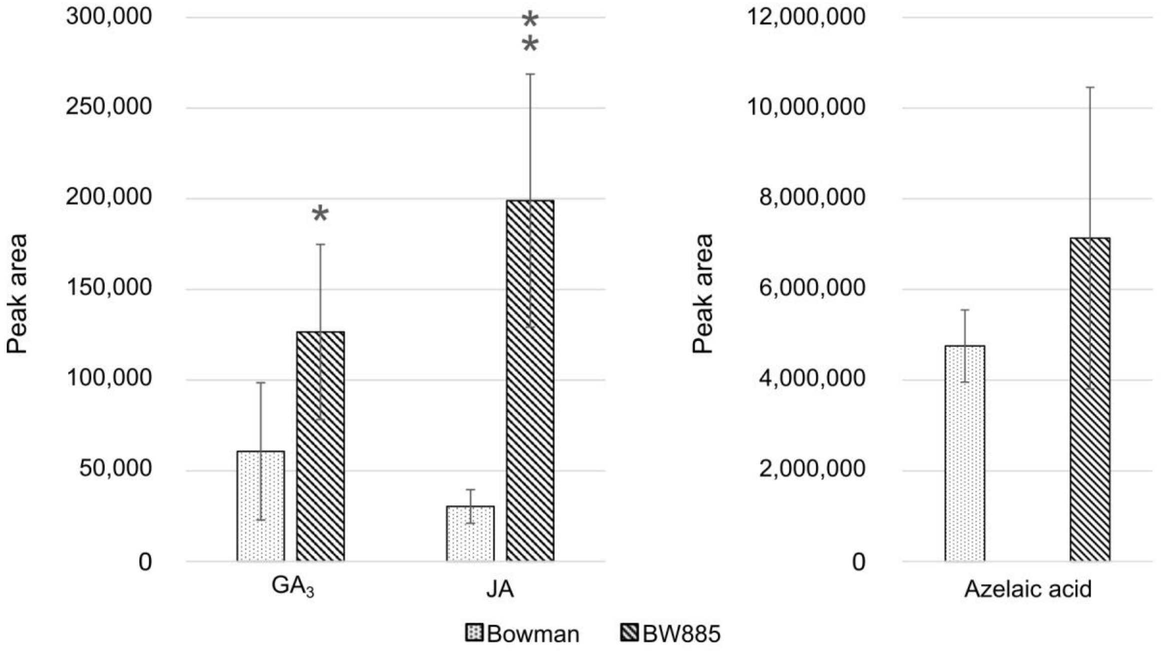

Supplementary Figure S5. Targeted analysis of gibberellin A_3 (GA_3), jasmonic acid (JA) and azelaic acid in BW885 brassinosteroid insensitive mutant by UHPLC-MS/MS. The analysis was performed on two weeks old plantlets, with 7 replicates for each line. Statistical analysis was done using a Wilcoxon rank sum test, *p*-value = 0,006993 (single asterisk) and 0,0005828 (double asterisk). This pathogen resistant and brassinosteroid insensitive line shows GA_3 and JA levels that are statistically different from Bowman control line, and a slight increase in azelaic acid content.

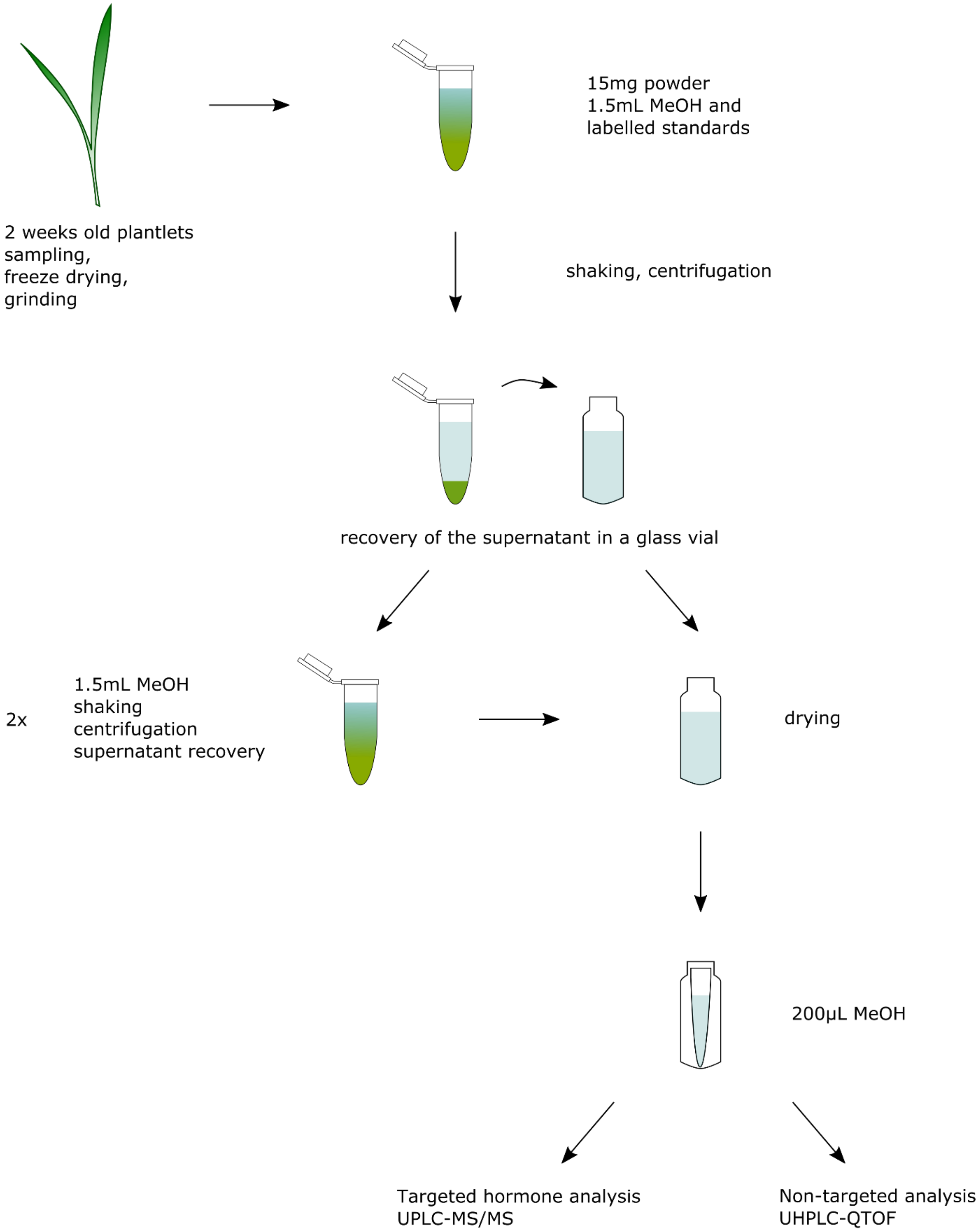

Supplementary Figure S6. Workflow of the extraction process.
